# Evidence-Based Management of Sickle Cell Disease: Ethnobotanical Survey and Laboratory Validation of Traditional Herbal Recipes

**DOI:** 10.3390/molecules31071192

**Published:** 2026-04-03

**Authors:** Marguerite Borive Amani, Mavar Manga Hélène, Mouithys Mickalad Ange, Nsasi Bakiantima Elodie, Ndezu Angirio Rachel, Memvanga Bondo Patrick, Batina Agasa Salomon, Marini Djang’eing’a Roland

**Affiliations:** 1 Department of Galenic Pharmacy and Drug Analysis, Faculty of Pharmaceutical Sciences, University of Kisangani, Kisangani P.O. Box 2012, Democratic Republic of the Congo; angiriorachel@gmail.com; 2 Laboratory of Pharmaceutical Analytical Chemistry, Department of Pharmacy, CIRM, University of Liege (ULiège), 4000 Liège, Belgium; 3 Department of Pharmacognosy, Faculty of Pharmaceutical Sciences, University of Kisangani, Kisangani P.O. Box 2012, Democratic Republic of the Congo; 4 Center for Oxygen Research and Development—CIRM, University of Liege, 4000 Liège, Belgium; 5 Centre de Recherche et d’Innovation Technologique en Environnement et en Sciences de la Santé (CRITESS), Faculty of Pharmaceutical Sciences, University of Kinshasa, Kinshasa P.O. Box 212, Democratic Republic of the Congo; 6 Department of Internal Medicine, Faculty of Medicine, University of Kisangani, Kisangani P.O. Box 2012, Democratic Republic of the Congo

**Keywords:** medicinal plants, soil type, additives, antioxidant, myeloperoxidase, sickle cell disease

## Abstract

In traditional medicine, laboratory tests are essential tools used to evaluate practices in healthcare that use natural products, particularly when there are few established treatments such as in the case of chronic diseases like sickle cell disease (SCD). SCD is a genetic hemoglobin disorder associated with hemolysis, oxidative stress, inflammation, and vaso-occlusive complications. This study aims to document medicinal plants used in the management of SCD and the traditional practices associated with their use, in order to assess their added value in relation to biological activities relevant to SCD. First, this study carried out an ethnobotanical survey at Kisangani, with 384 participants. A total of 201 respondents, 58% of whom were women, identified 45 medicinal plant species from 30 families. They reported the use of sugar, caramel, ash, and lemon juice as additives in traditional remedies. Based on several criteria, *Alchornea cordifolia* Müll. Arg. and *Hibiscus tiliaceus* L. were selected for laboratory evaluation. Their leaves were collected from arid and marshy soils and prepared according to the respondents’ descriptions for antioxidant and anti-inflammatory assessments. *A. cordifolia* showed significantly higher activities than *H. tiliaceus* regarding antioxidant (*p* = 0.001) and anti-inflammatory (*p* = 0.01) properties. Soil type was observed to influence the bioactivity of *H. tiliaceus*, with better performance in marshy soil samples. Sugar and caramel were found to reduce antioxidant activity, whereas ash and lemon juice preserved it without markedly altering anti-inflammatory effects. These findings are promising for supporting local knowledge about these two plant species.

## 1. Introduction

The use of traditional medicinal plants requires evidence-based validation, particularly for chronic diseases such as sickle cell disease (SCD). SCD is the most prevalent hemoglobinopathy worldwide and a major cause of morbidity and mortality [1]. This is because it causes the substitution of a single amino acid in the β-globin chain, which leads to hemoglobin polymerization, intravascular hemolysis, and the release of free hemoglobin and heme. These events trigger oxidative stress and inflammation through myeloperoxidase (MPO) activation, reactive oxygen species (ROS) generation, and neutrophil extracellular trap (NET) formation, contributing to vaso-occlusion and organ damage [2,3]. SCD is characterized by chronic anemia, vaso-occlusive crises, acute chest syndrome, stroke, renal impairment, and splenic dysfunction [4,5], resulting in a long-term family burden. 

As mentioned, this disease affects millions of people worldwide, with approximately 300,000 new cases per year, predominantly in Sub-Saharan Africa, the Middle East, and India, although migration contributes to its global spread [6]. In Africa, around one thousand children are born daily with SCD, and more than half die before five years of age due to infections and severe anemia [7]. In the Democratic Republic of the Congo (DRC), SCD prevalence is about 2% among newborns, placing the country as the second most affected African country after Nigeria [8], with nearly 50,000 annually affected births and high early mortality [9]. In Kisangani, the homozygous prevalence among newborns is 2.2% while, for heterozygotes, it remains at 21%, which is quite stable [10,11]. 

The therapeutic strategy for SCD management involves preventing hemoglobin S polymerization by administering agents that induce or increase fetal hemoglobin (HbF) production [12]. Another promising therapeutic strategy involves targeting the gene responsible for the mutation through gene therapy, in order to correct or compensate for the defective β-globin gene [13]. However, the high cost and limited accessibility of these therapeutic strategies in many developing countries often compel patients to rely on conventional medicines or medicinal plants for the management of SCD symptoms [14,15]. The high prevalence of SCD, the difficulties associated with its management, and the social stigma experienced by affected individuals have contributed to its recognition as a neglected tropical disease [16,17].

Several studies have identified African plants commonly used in traditional medicine for the management of SCD [18,19,20,21], for which numerous studies have been carried out to evaluate their biological activities relevant to this condition. These studies have primarily focused on antisickling, anti-inflammatory, and antioxidant activities, investigated using either crude extracts or metabolites isolated from these plants [22,23,24,25,26,27,28].

Several improved traditional medicines from medicinal plants, such as Niprissan, Faca, and Drepanoalpha, have been developed and standardized in some countries to improve SCD management [29,30].

However, most of these studies have focused on crude extracts or isolated metabolites, while relatively little attention has been paid to the traditional practices associated with the use of these plants.

**Figure 1:**
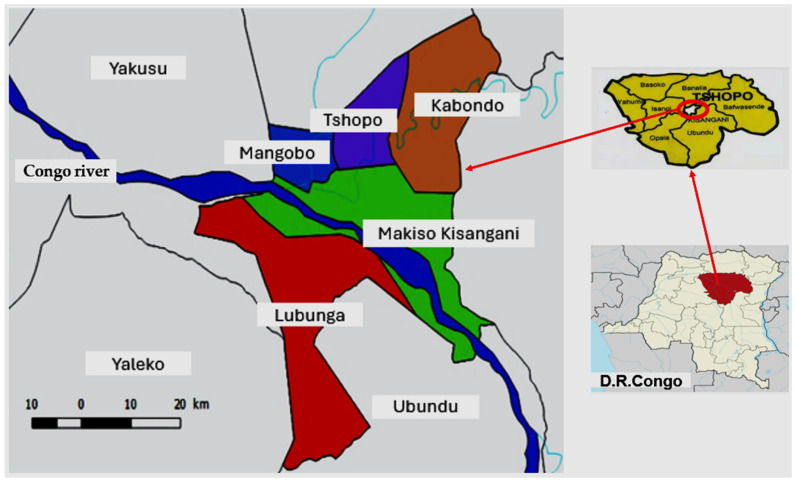
Map of Kisangani city crossed by the Congo river.

Therefore, given the high prevalence of SCD in Kisangani and the predominant reliance on medicinal plants for its management according to local perceptions and practices, an ethnobotanical survey was conducted in this city (Figure 1) to document the medicinal plants used and their associated modalities of use. Based on these findings, some species were selected and submitted for laboratory investigation to assess the added values of local practices to the biological activities relevant to SCD.

## 2. Results and Discussions

### 2.1. Ethnobotanical Survey Outcomes

#### 2.1.1. Characteristics of Respondents

Using a questionnaire developed in accordance with ethical principles, the survey was conducted in the six municipalities of Kisangani through direct interviews. Respondents were approached either at their homes or workplaces or, in some cases, in public places such as streets.

Out of 384 participants, 201 reported using medicinal plants for the management of SCD. Women (58.7%) represented more than half of the respondents, which can be explained by their key role in caregiving and health management, particularly with children with chronic diseases, including SCD. Sociocultural perceptions of such disease often attribute care responsibilities to women.

Another notable fact regarding this survey is that most of the participants were educated, older than 20 years of age, and felt free to claim being affected by SCD, either closely or indirectly, which supports the reliability of their responses. In addition, their free participation and diverse knowledge sources indicate that SCD acceptability and sociocultural perception among people has evolved compared to 2007, when patients were victimized and rejected by society [31].

The main knowledge sources were from friends (39%), family (23%), and school and university (15%). Media and churches were rarely cited, while some participants were not clear about this point.

Respondents were classified into three groups: patients (12.9%) were recruited through the sickle cell patients’ association in Kisangani; healthcare professionals (28.8%) included physicians, pharmacists, and nurses familiar with the use of medicinal plants; and the general population (58.2%) comprised men and women from various occupational backgrounds (Table 1).

#### 2.1.2. Plants Species Cited by Respondents and Their Way of Use

The respondents reported the use of 45 distinct plant species in Kisangani for SCD treatment. As shown in Table 2, 25 species were cited at least thrice by respondents, indicating high usefulness. Seven species were mentioned once by two respondents and, notably, thirteen species were mentioned by a single participant. The participants mentioned that they learned about some plants serendipitously and others by knowledge-sharing with traditional healers, grandparents, or friends. Even if they could not explain the biological mechanism of action of the plant recipes, the participants testified to their favorable results, such as the loss of pain and increases in hemoglobin levels. The plants were identified by their vernacular name and were assigned a deposit number from the Faculty of Science of Kisangani University along with the family.

We also classified the plants according to their use report (UR) number, which includes the number of ways a plant is used. For example, *H. tiliaceus* had 22 URs, meaning that 42 respondents described 22 ways of using that plant, which is the highest UR value and confirms its importance for SCD management.

Concerning the used parts for all plants, none of the participants could justify their answer. However, we noticed that leaves (fresh) were the most used as they are easily available. In some cases, respondents recommended using leaves that had dried naturally on the tree (e.g., *Carica papaya*, *Musa paradisiaca*, *Alchornea cordifolia*, *Theobroma cacao*), although the rationale underlying this practice was not clearly explained. Additionally, no participants could justify the requirements concerning the dry or fresh state of used parts, or the harvesting requirements, including the type of soil. Note that Kisangani city mainly has arid or marshy soils, which can influence the recipes’ composition. Some respondents believe that plants grown in marshy soil would be more effective. This was confirmed by riverside communities; specifically, the “Genya”, who live on the Congo river in Kisangani, testified to employing water for spiritual deliverance and healing. Decoction was cited as the most used preparation method, with 108 URs (58.6%), followed by maceration (20.1%).

The oral route was the most frequently cited for administration with 154 URs (83.6%), since it is the easiest controllable route. However, the cutaneous and rectal routes, mentioned 10 and 3 times, respectively, caught our attention as they are little known routes of administration for diseases such as SCD. According to some practitioners, the cutaneous administration route is appropriate because it alludes to a customary purification ceremony, given that SCD is considered a curse. Thus, this deserves further investigation to assess both routes’ therapeutic value.

#### 2.1.3. Frequency of Citation of Additives 

**Figure 2:**
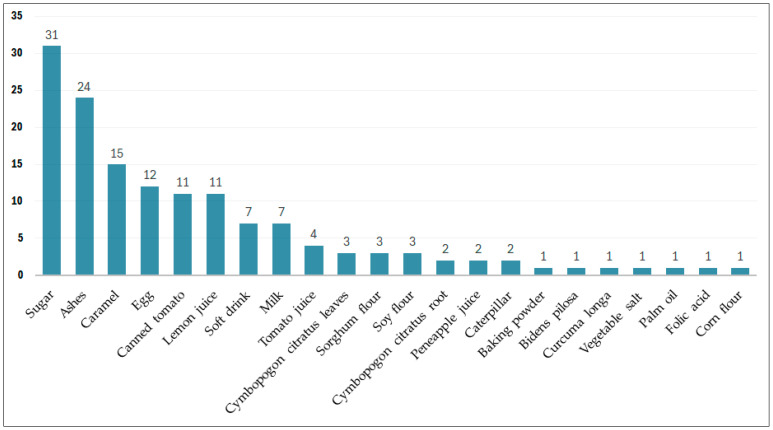
Frequency of additive citations.

Of the 184 URs, 111 involved a combination of plant extracts with other products (Figure 2). The most frequently associated products included sugar, ash, caramel, canned tomatoes, lemon juice, and eggs. In addition, some participants indicated that additives such as canned tomatoes, milk, and eggs should be used separately from some herbal recipes.

The following additives are typically added during the preparation of herbal remedies: caramel, sugar, lemon juice, and ash. Meanwhile, caterpillars, sorghum, maize, and soya flour are consumed with the meal after the ingestion of the herbal remedy. We found that the key difference was in the pre-treatment of certain additional products before their incorporation into the recipe, as in the case of sugar calcination or *C. citratus* roots. In other cases—for example, in topical applications—the associated products are consumed orally after external administration of the principal product. Unfortunately, the participants were unable to explain the benefits of such combinations and administration routes. Most responses were limited to describing the organoleptic changes that occur during the preparations after the addition of these products. Other participants claimed that the tonic effects of these additives act against anemia-related fatigue or as appetite stimulants.

Note that, in most African cultures, ash is associated with purification, transition, and renewal, whereas fire denotes destruction, which then allows for regeneration. Its inclusion in remedies therefore symbolizes the transition from illness to healing. Lemon is often seen as a cleansing agent, as a "hunter of evil spirits." Its acidity is perceived as a force capable of fighting disease or removing “impurities” from the body [32,33,34]. Some informants believed that these additives are used for not only their antiseptic and preservative properties but also their ability to alter the taste of the remedy, thus facilitating its administration. From a scientific point of view, the addition of lemon is beneficial because of the ascorbic acid, which may contribute to Fe(III) reduction to Fe(II), facilitating its oral absorption and subsequently increasing hemoglobin levels [35].

Data obtained from the ethnobotanical survey were used to design the flowchart guiding the selection of plant species for laboratory evaluation. Particular emphasis was placed on the soil type criterion, considering the geographical context of Kisangani, a city located on islands within the Congo River basin and surrounded by equatorial forest ecosystems where vegetation develops under diverse soil conditions, ranging from marshy to relatively dry soils. This context prompted our investigation into the potential influence of soil type on the biological properties of the selected plants.

In addition, special attention was paid to the additives incorporated during the preparation of traditional remedies. These substances may remain in contact with plant metabolites for a certain period of time, potentially inducing chemical transformations that could modify the chemical composition of the extracts and, consequently, their biological activities.

### 2.2. Laboratory Validation

#### 2.2.1. Plant Selection for Laboratory Assessment

We used the UR value for plant selection. Recall that a high UR for a plant indicates that several variants were mentioned by the participants, either in terms of used parts, preparation methods, or administration routes. In our survey, the top five species most frequently mentioned were *H. tiliaceus*, *P. americana*, *C. papaya*, *T. grandis*, and *C. sulphureus*.

However, we considered additional selection criteria such as the local species availability, previous studies related to SCD, the soil type specificity mentioned by the respondents, the administration route, and the types of additives associated with the preparation. Thus, we included only plants for which the most cited additives (sugar, ash, caramel, and lemon juice) were explicitly mentioned at the time of recipe preparation. For example, eggs were not included because they are not incorporated during preparation, but administered sometimes after the remedy has been ingested.

Among the above top five species, *P. americana* and *C. papaya* were excluded since they were not mentioned for both soil types, *T. grandis* was excluded because it is not administered orally, and *C. sulphureus* did not meet the criterion related to the use of the most reported additives. In addition to the previously described criteria, recommendations regarding the type of leaves to be used were also reported, particularly leaves that had dried naturally on the tree.

**Figure 3:**
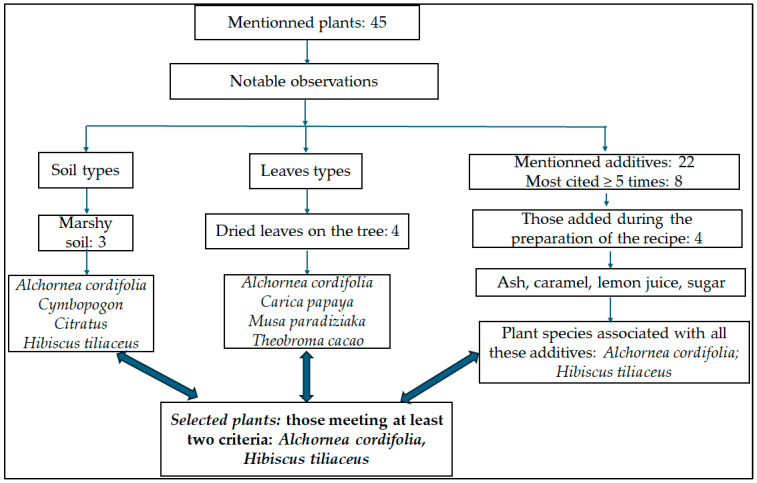
Flowchart describing the plant selection process.

Considering the three main criteria (Figure 3), plant species fulfilling at least two of these criteria were selected. As a result, *H. tiliaceus* and *A. cordifolia* were obtained for laboratory evaluation. AC is a shrub belonging to the Euphorbiaceae family, commonly found in tropical forests, especially along rivers. HT is a tree from the Malvaceae family, found in tropical regions of Africa, America, and Asia, which is more drought-tolerant and adaptable to different soil types.

#### 2.2.2. Evaluation of Biological Activities 

We evaluated the antioxidant and anti-inflammatory effects of the selected plants and the added value of the additives reported by respondents. Oxidative stress plays a prominent role in SCD, which leads to several complications [36,37]. Thus, anti-inflammatory activity was assessed using both the classical myeloperoxidase (MPO) assay and the SIEFED (Specific Immuno-Extraction Followed by Enzymatic Detection) method, given the role of MPO in SCD-related oxidative stress and inflammation. In addition to its peroxidase activity, MPO catalyzes chlorination reactions in the presence of chloride ions [38]. The classical MPO assay evaluates the direct inhibitory effect of plant extracts on enzyme activity in an open system, whereas the SIEFED assay selectively measures the activity of active, bound MPO, minimizing interference from other extract constituents [39,40]. The HT plant was already subjected to chemical characterization in our previous study (41); in this study, antioxidant activity was assessed using ABTS radical cations.

##### Antioxidant Activity

As shown in Table 3, all AC extracts presented higher and significant antioxidant activity than their corresponding HT extracts, regardless the soil type with or without additives. This indicates that antioxidant activity is species- and concentration-dependent. For AC, we noticed that arid soil had overall higher antioxidant activity than marshy soil. This finding is opposite to that for HT, as marshy soils were more favorable. However, regarding AC extracts and additives, the use of lemon juice was found to be favorable since the antioxidant activity was higher compared to the use of ash, caramel, and sugar additives. In the case of AC extracts, lemon juice and ash were found to be favorable since the antioxidant activity was higher compared to sugar and caramel. These antioxidant activity patterns were confirmed through ABTS tests. Overall, none of these additives significantly improved the antioxidant capacity of the extracts, which might be due to an interference or dilution effect of the phytochemical compounds. The antioxidant activity observed in the total extracts of the two plants supports the satisfaction expressed by recipients, according to survey responses, as well as its use as a complementary strategy of SCD management. Furthermore, the antioxidant activity of AC and HT extracts has been confirmed in previous studies [41,42]. 

##### Anti-Inflammatory Activity

**Figure 4:**
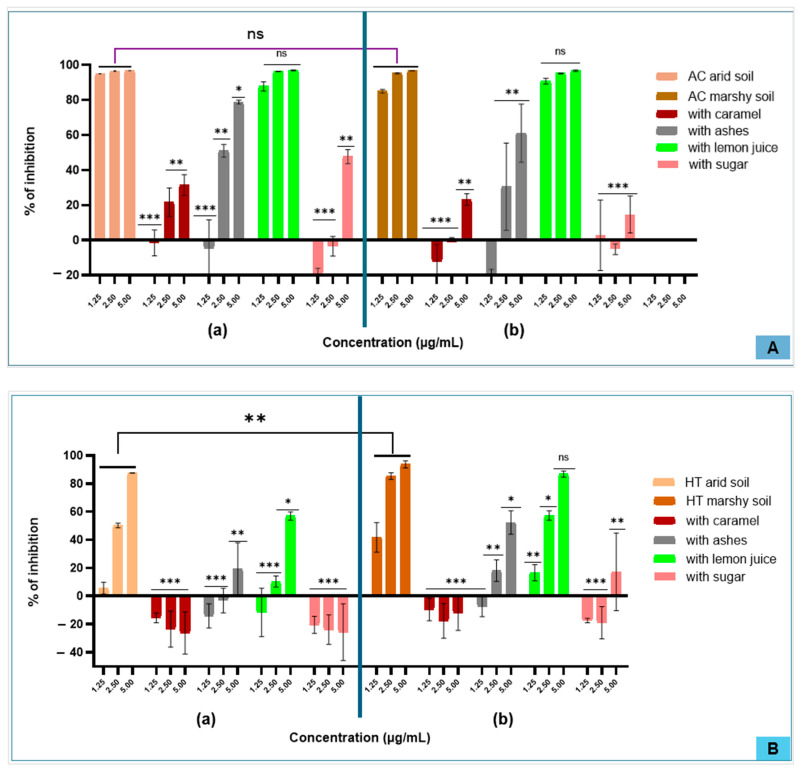
Anti-inflammatory profiles expressed as the percentage inhibition of classical myeloperoxidase (MPO) activity for *Alchornea cordifolia* (AC) (**A**) and *Hibiscus tiliaceus* (HT) (**B**). Extracts obtained from plants growing in arid soil (**A**(**a**),**B**(**a**)) and marshy soil (**A**(**b**),**B**(**b**)) and tested as aqueous extracts alone or in combination with additives, ((ns): *p* > 0.05; (*): *p* < 0.05; (**): *p* < 0.004; (***): *p* < 0.001).

**Figure 5:**
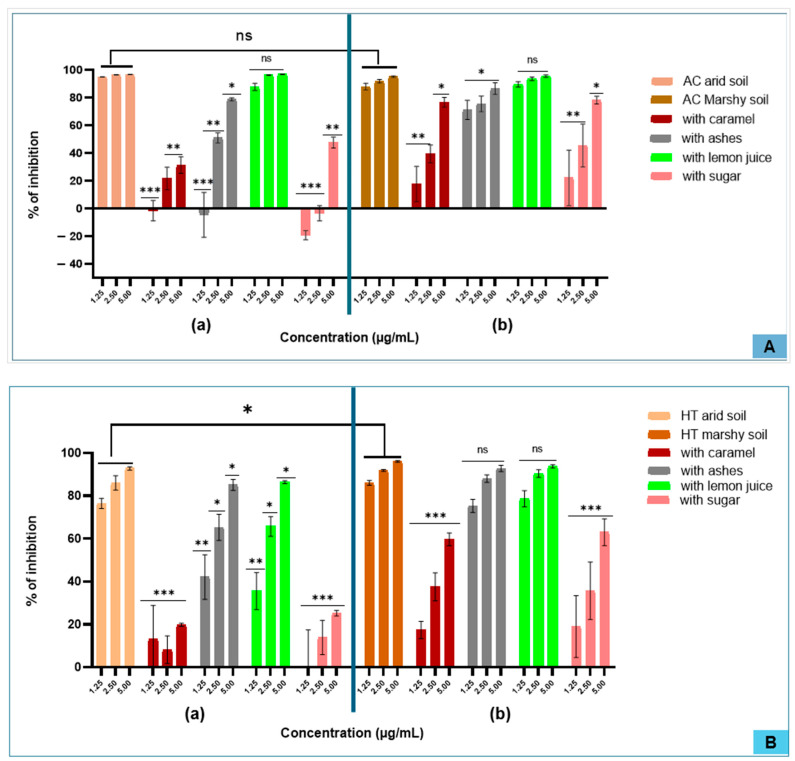
Anti-inflammatory profiles expressed as the percentage inhibition of SIEFED activity for *Alchornea cordifolia* (AC) (**A**) and *Hibiscus tiliaceus* (HT) (B). Extracts obtained from plants growing in arid soil (**A**(**a**),**B**(**a**)) and marshy soil ((**A**(**b**),**B**(**b**)) and tested as aqueous extracts alone or in combination with additives, ((ns): *p* > 0.05; (*): *p* < 0.05; (**): *p* < 0.004; (***): *p* < 0.001).

In terms of the inhibition of human MPO activity, the AC extract presented high activity compared to the HT extract. Among the tested additives, only the extract treated with lemon juice exhibited high activity, reaching approximately 90% for AC compared to 60% for HT at a concentration of 5 µg/mL. This level of inhibition is comparable to that of the untreated extract of AC, but remains lower than that observed with the untreated extract of HT. Furthermore, the HT extract from marshy soils showed greater inhibitory activity than that from arid soils. However, for AC, soil type did not appear to significantly influence this activity. A concentration response dependence was observed with HT extracts, whereas AC extracts quickly reached an inhibition plateau at low concentrations, suggesting a saturation effect (Figure 4 and Figure 5).

Classical MPO tests

SIEFED tests

These results suggest that the extracts tested inhibit MPO activity by preventing the enzyme from interacting with its substrates, particularly hydrogen peroxide (H_2_O_2_) and nitrite ion (NO_2_^−^). This inhibition limits the formation of reactive oxygen species (ROS), which are responsible for oxidative stress and inflammatory lesions [43].

Regarding myeloperoxidase (MPO) inhibition, this study shows that among the four additives tested, the incorporation of ash or lemon juice did not significantly modify the inhibitory activity of extracts from either plant. Inhibition levels ranged from 90 to 100% at 5 µg/mL, with no significant difference compared to untreated extracts. Similarly, soil type exerted a negligible effect on MPO inhibition for both AC and HT. Unlike the classical MPO assay, which primarily measures inhibition through interference with reactive oxygen species (ROS) formation via electron or hydrogen transfer mechanisms, the SIEFED assay specifically assesses the spatial interaction between test molecules and the enzyme’s active site. This approach, which focuses on the molecular arrangement, may explain some of the variations or unexpected results observed in inhibitory activity [39]. The anti-inflammatory activity observed for AC confirms the results reported in previous studies [42,44]. The same is true for HT, whose antioxidant and anti-inflammatory properties are well documented [45]. Several studies have also highlighted the immunomodulatory and thrombolytic effects of HT [46,47] and antibacterial effects of AC [48,49]. 

#### 2.2.3. Phytochemical Composition of Selected Plant Species

The leaves of *A. cordifolia* have been reported to contain phenolic acids, including gallic acid and ellagic acid, flavonoids such as vitexin, rutin, quercetin, myricetin, quercitrin, kaempferol, naringenin, and hyperin, alkaloids (e.g., yohimbine and alchorneine) and terpenoids such as friedelin, bisabolol, and linalool [50,51,52]. Similarly, *H. tiliaceus* leaves have been reported to exhibit a diverse phytochemical composition, including flavonoids such as apigenin, isoquercitrin, astragalin, rutin, transtiliroside, kaempferol, and quercetin, as well as phenolic acids, triterpenoids (e.g., friedelin), alkaloids, tannins, and megastigmanes such as tiliacic acid [45,53,54,55].

## 3. Materials and Methods

### 3.1. Material

#### 3.1.1. Survey Area and Period

The survey took place in Kisangani city from June 2023 to December 2024. The city is in the north-east of the DRC, very close to the equatorial line and surrounded by a dense tropical rainforest zone. The climate is equatorial with a short, hot, dry season, mainly in January and February, followed by a warm, oppressive, and overcast rainy season that persists throughout most of the year.

#### 3.1.2. Vegetable Materials and Reagents for Bioassays

##### Vegetable Materials

The plant materials consisted of leaves from the two plants species selected based on the ethnopharmacological survey. The leaves were harvested from plants growing on marshy and arid soils, and the leaf extracts were prepared following the respondents’ descriptions, with or without the additives mentioned during the survey.

##### Reagents

All salts used to prepare the buffered solutions and methanol were of analytical grade, obtained from Merck VWR (Leuven, Belgium). 2,2 azino-bis(3-ethylbenzothiazoline)-6-sulfonic acid (ABTS), Amplex Red, sodium nitrite, sodium persulfate, and hydrogen peroxide (H_2_O_2_) were purchased from Sigma-Aldrich (Steinheim, Germany). Bovine serum albumin (BSA) was obtained from Roche Diagnostics Gmbh (Mannheim, Germany), and human MPO was purchased from Calbio chem Millipore (Bellirica, Madison, WI, USA). Ash was obtained from the charcoal of equatorial rainforest wood, sugar from sugarcane (*Saccharum* spp.), and lemon juice from yellow lemons of the *Citrus limonia* (L.) Osbeck species.

### 3.2. Methods

#### 3.2.1. Ethnobotanical Survey

The survey team included eight pharmacy students and two assistants from the Department of Pharmacy, Faculty of Medicine and Pharmacy, at the University of Kisangani. An anthropologist was also recruited to ensure full understanding of the responses during the survey. The sample size of respondents (*n*) was estimated using William Cochran’s formula, as described by Charan J. (Equation (1)) [56,57]:

(1) $$n=\frac{z^{2}\times p\times (1-p)}{m^{2}}$$

where *z* is the confidence level (1.96 at 95%); *p* is the estimated proportion of the population with the target characteristic; and *m* is the tolerated margin of error. When set at 5%, *m* equals 0.05.

Respondents were selected based on two criteria: being at least 18 years old and having resided in Kisangani for a minimum of 3 years. The objectives of the survey were explained to the participants. Their voluntary, free, and informed consent was obtained in accordance with ethical guidelines for research involving human subjects. Data were then collected using a semi-structured questionnaire focusing on knowledge and practices related to SCD management, particularly traditional medical approaches. Information gathered included plant names, the parts used, remedy preparation, administration routes, and specificity and individual perceptions of therapeutic efficacy, including plant combinations. The cited plant species were identified at the Faculty of Science, University of Kisangani, and taxonomically verified using the African Plant Database and World Flora Online.

#### 3.2.2. Laboratory Assessment

##### Plant Selection and Collection

The plant species were sorted based on the survey data and the following selection criteria: (i) the frequency of citations by respondents; (ii) the leaf type; (iii) the route of administration being limited to oral use; (iv) the harvesting environment, considering both arid and marshy soils for the same species; and (v) and the additives used during recipe preparation, focusing on the four most frequently reported additives associated with each plant. Practical considerations included local availability and evidence from previous studies reporting a link between their medicinal properties and SCD management. The parts used were collected from the selected plants growing on two different soils (marshy and arid environments), then air-dried at room temperature in the laboratory of the Faculty of Medicine and Pharmacy, University of Kisangani. The dried plant material was subsequently ground into powder using a ZM 200 ultra-centrifugal electric grinder (Retsch, Haan, Germany). The resulting powders were packaged in hermetically sealed plastic vials and transported to the University of Liège for further analyses at the Pharmacognosy Laboratory (LPG) and the Oxygen Research and Development Centre (CORD).

##### Preparation of Plant Extracts

The selected and collected plants were treated with the most cited additives. The untreated raw extracts of each plant from arid soils were used as controls. These were compared with the plant extracts from marshy soils to provide evidence supporting the respondents’ statements.

For each selected plant, 25 g of powder was mixed into 500 mL of water. The mixture was boiled at 100 °C for 15 min and then filtered through glass wool. The quantities of additives to be incorporated were determined based on the proportions reported by the respondents (2.0 g of ash, 2.5 mL of lemon juice, and 10.0 g of sugar per 100 mL of aqueous extract). Regarding caramel, respondents said to add an amount equivalent to 10 g of sugar per 100 mL of extract, but only after the sugar had caramelized prior to mixing with the decoction. In order to reproduce the traditional preparation method described by the respondents, 100 mL of hot aqueous extract was put into separate beakers, and one additive was added to each of them. The resulting mixtures obtained were allowed to cool to room temperature and were then filtered through glass wool and freeze-dried for 48 h. The resulting dry extracts were transferred into glass vials and stored at 6 °C until use in subsequent biological assays.

##### Biological Activity Assays

Antioxidant activity

Antioxidant activity was assessed using the ABTS test to obtain a more comprehensive assessment of the antioxidant capacity of the different samples [58]. The ABTS test is based on the change in the blue-green color of the ABTS radical cation (ABTS^•+^) solution into its colorless neutral form, as previously described by Re et al. and modified by Widowati et al. [59,60]. To generate ABTS^•+^ radicals, an aqueous solution of sodium persulfate (2.45 mM) was mixed with ABTS (7 mM) and incubated overnight in the dark to obtain a dark-colored solution. The stock solution of ABTS^•+^ was then diluted by adding pure methanol (100%) to obtain an absorbance of 0.70 (±0.02) at 734 nm at 25 °C. Assays were performed in multiwell plates (*n* = 3, *N* = 2). An aliquot of 2 µL of the tested extract was added to 198 µL of ABTS^•+^. As a negative control, 2 µL of ultrapure water was added to 198 µL of ABTS^•+^ solution. To evaluate the absorbance of the different solutions at 734 nm, a microplate reader (Thermo Lab system, Vantaa, Finland) was used and the reducing capacity was determined according to the following formula:

%inhibition = (A control − A sample) ∗ 100/A control(2)

where A is the absorbance.

Anti-inflammatory activity

The peroxidase activity of MPO was measured using a classical enzymatic assay and the SIEFED assay, as described by Nyssen et al. [61]. The MPO solution was prepared with purified human MPO in dilution buffer (PBS 20 mM at pH 7.4 with 5 g/L BSA and 0.1% Tween-20). Solutions of each sample, at final concentrations ranging from 1.25 to 5.00 µg/mL, were incubated with human MPO at a final concentration of 5 mU/mL for 10 min before further use. MPO activity was determined by monitoring the enzyme-catalyzed oxidation of Amplex Red in the presence of H_2_O_2_ and nitrite in phosphate buffer at pH 7.4.

Classical assay of MPO activity

After incubation, mixtures containing 100 µL of each extract or vehicle (ultrapure water) and MPO were placed on a multiwell plate (*n* = 3, *N* = 2), and peroxidase activity was measured by adding 10 µL of sodium nitrite solution (4.5 mM, final concentration) and 100 µL of the reaction solution containing 10 µM H_2_O_2_ and 40 µM Amplex^®^ Red (AR) in phosphate buffer (50 mM) at pH 7.4. The oxidation of AR to the fluorescent resorufin adduct (excitation = 544 nm; emission = 590 nm) was monitored for 30 min at 37 °C using a fluorescence plate reader (Fluoroskan Ascent, Fisher Scientific, Hampton, NH, USA).

SIEFED assay of MPO activity

Samples containing MPO and different concentrations of juglone were prepared and incubated as in the classical assay. Then, 100 µL of each mixture (MPO alone or MPO+ extract) was placed on a SIEFED multiwell plate coated with rabbit polyclonal antibodies (3 g/mL) against human MPO and incubated for 2 h at 37 °C in the dark (*n* = 3, *N* = 2). After washing the wells, the activity of the enzyme captured by the antibodies was measured by adding 10 µL of sodium nitrite solution (4.5 mM, final concentration) and 100 µL of a reaction solution containing 10 µM H_2_O_2_ and 40 µM Amplex^®^ in phosphate buffer (50 mM) at pH 7.4. The oxidation of Amplex^®^ Red to the fluorescent adduct resorufin (excitation = 544 nm; emission = 590 nm) was monitored for 30 min at 37 °C using a fluorescence plate reader (Fluoroskan Ascent, Fisher Scientific). As for the direct MPO assay, a control assay, set as a relative value of MPO activity, was performed with purified MPO in the presence of PBS instead of the samples. In this SIEFED assay, MPO was bound to the wells by the antibodies, and the test extract was discarded in the wash step before the enzymatic activity was measured. For both MPO assays, the percentage inhibition was calculated using a formula similar to that described above (see Equation (2)).

##### Statistical Analysis

For data analysis, we performed statistical analysis with the GraphPad Prism 8.0.1 software, developed by GraphPad (San Diego, CA, USA). Two-way ANOVA multiple comparisons and Dunnett’s post-tests were used to test for differences between treatment groups, and results were considered significant at *p*-values of less than 0.05; i.e., at the 95% confidence level.

## 4. Conclusions

The management of sickle cell disease (SCD) in Kisangani remains a major public health challenge, leading many families with limited resources to rely on traditional medicine, particularly medicinal plants combined with various additives such as ash, sugar, caramel, and lemon juice. Laboratory evaluation of two frequently used species (*A. cordifolia* and *H. tiliaceus*) confirmed their antioxidant and anti-inflammatory activities, which may partly explain the positive experiences reported by users. Notably, the addition of ash and lemon juice reduced antioxidant activity without markedly affecting anti-inflammatory effects, whereas sugar and caramel significantly impaired both activities. In addition, soil type influenced plant bioactivity in a species- and activity-dependent manner. Future in vivo investigations should broaden the evaluation of biological activities and safety parameters to provide stronger evidence-based guidance, while advanced analytical techniques such as HPLC–MS should be utilized to identify and characterize the chemical constituents involved.

## Figures and Tables

**Table 1:** Socio-demographic profile of respondents.

Sample Characteristics	*N* (201)	%
Age of respondents (years)		
18–35	89	44.3
36–50	84	41.8
50 and over	28	13.9
Gender		
Women	118	58.7
Men	83	41.3
Respondents’ categorization		
Healthcare professionals	26	12.9
Sickle cell Patients	58	28.9
General population	117	58.2
Respondents Hemoglobinic status		
AA	58	28.9
AS	28	13.9
SS	30	14.9
Unknown	85	42.3
Circumstances of knowledge		
Church	1	0.5
Family	46	22.9
School/University	30	14.9
Friendship	78	38.8
Media	8	4.0
Hospital	38	18.9

**Table 2:** Plants mentioned and use reports of each species cited.

Deposit Number	Plant Names	VN Cited by Participants	Family	Used Parts	Preparation Method	Administration Route	Combination	FC	UR
MBA/01	*Acmella paniculata* (Wall. ex DC) R.K.Jansen	Kekemu	Asteraceae	WP, FL	Dec	Rectal, Oral	nd	2	2
MBA/05	*Alchornea cordifolia* (Schumach.) Müll.Arg.	Mabanzi	Euphorbiaceae	FL, DLT	Inf, Dec, Mac	Oral	ash, caramel, lemon juice canned tomato, sugar	17	3
nd	*Amaranthus cruentus* L.	Muchicha	Amaranthaceae	FL	Cooking	Oral	sugar, lemon juice	3	3
nd	*Ananas comosus* (L.) Merr.	Anana	Bromeliaceae	Fruit	Mac, EJ, Dec	Oral	canned tomato, soft drink, sugar, eggs, caramel	7	6
MBA/25	*Andasonia digitata* L.	Liguma	Malvaceae	FL, DF	Dec, Mac	Oral	sugar, milk, eggs, folic acid, caramel, milk+ eggs, eggs+ sugar	8	6
MBA/35	*Anisopappus chinensis* Hook.& Arn.	Nzete ya makila	Asteraceae	FL, Flower	Mac, Dec	Oral, Cutaneous	ash	5	4
MBA/28	*Annona reticulata* L.	Bizabibu	Annonaceae	FL	Dec	Oral	ash	1	1
nd	*Annonidium manii* (Oliv.)	Nzete ya bombi	Annonaceae	FL, Bark	Dec	Oral, Cutaneous	nd	4	2
MBA/6	*Bidens pilosa* L.	Police	Asteraceae	WP	Dec	Oral	nd	1	1
MBA/20	*Bridelia atroviridis* Müll.Arg.	Mgiangange	Phyllanthaceae	FL	Dec	Oral	nd	1	1
MBA/07	*Carica papaya* L.	Payipayi	Caricaceae	FL, DLT	Dec, Mac	Oral, Cutaneous	*C.citratus* root, ash, caramel, pineapple juice, sugar, canned tomato, soy flour, caterpillar	14	13
MBA/23	*Catharanthus roseus* (L.) G. Don		Apocynaceae	Flower	Dec	Oral	sugar	1	1
MBA/11	*Cocos nucifera* L.	Cocoti	Arecaceae	Juice, FL	Mixt, Dec	Oral	soft drink, sugar	3	3
MBA/29	*Coffea robusta* L. Linden	Kafé	Rubiaceae	FL, Seed	Dec, Mac, Grinding	Oral	*C. citratus,* sugar, milk, eggs, caramel, ash	4	5
MBA/22	*Cosmos sulphureus* Cav.	Maloti	Asteraceae	Flower, leaves, root	Mac, EJ, Dec Grinding	Oral	ash, sugar	10	9
nd	*Cucurbita pepo* L.	Kasa ya maboke, djurubi	Cucurbitaceae	FL	EJ, Dec, Mac	Oral	sugar, ash, caramel	6	6
MBA/21	*Cymbopogon citratus* (DC.) Stapf	Nyasi	Poaceae	FL, Root	Inf, Dec	Oral	*B. pilosa* +sugar, *C. longa*	3	3
MBA/16	*Elaeis guineensis* Jacq.	Ngasi, Nzete ya lito	Arecaceae	Fruit	Mac	Rectal	nd	1	1
nd	*Fagara zanthoxyloides* (Lam.) B. Zepernick & Timler	-	Rutaceae	FL	Mac, Dec	Oral	sorghum powder	3	2
nd	*Ficus mucuso* Welw. Ex Ficalho	Apendanyoka	Moraceae	FL	Dec	Oral	nd	1	1
MBA/13	*Harungana madagascariensis* Lam.ex Poir.	Botondolondo	Hypericaceae	FL	Dec	Oral	sugar	2	2
MBA/33	*Hibiscus sabdariffa* L.	Ngai-ngai	Malvaceae	FL	Dec, Cooking	Oral	*C. citratus* leaves, sugar	7	5
MBA/30	*Hibiscus tiliaceus* L.	Kasa ya makila	Malvaceae	FL	Dec, EJ, Mac, Inf	Oral, Cutaneous	ash, sugar, soy flour, milk corn flour, tomato juice, soft drink, lemon juice, caterpillar, *C. citratus*, caramel,	42	22
MBA/26	*Ipomoea batata* (L.) Lam.	Matembela	Convolvulaceae	FL	Mac	Oral	tomato juice, eggs, sugar, milk	2	2
MBA/04	*Laportea canadensis* Wedd.	Ibenja, Katolia	Urticaceae	WP	Dec	Rectal, Oral	nd	3	2
MBA/19	*Macaranga spinosa* Müll.Arg.	-	Euphorbiaceae	FL	Dec	Oral	nd	1	1
MBA/17	*Macaranga stipulosa* Müll. Arg.	-	Euphorbiaceae	FL	Dec	Oral	nd	1	1
MBA/34	*Mangifera indica* L.	Manga	Anacardiaceae	FL	Dec	Oral	eggs	1	1
nd	*Manihot esculenta* Crantz	Sombe, Mwinja	Euphorbiaceae	FL	Dec, Inf, Mac, Cooking	Oral	lemon juice, milk	8	7
MBA/10	*Morinda morindoides* (Baker) Milne-Redh.	Kongo bololo	Rubiaceae	FL	Inf	Oral	nd	1	2
MBA/08	*Moringa oleifera* Lam.	Moringa	Moringaceae	FL	Mac, Dec, Inf	Oral	sugar	8	4
MBA/24	*Musa paradisiaca* L.	Makemba	Musaceae	DLT	Dec	Cutaneous, Oral	ash, sugar	4	3
MBA/09	*Myrianthus arboreus* P. Beauv.	Bokomu	Moraceae	FL	Dec, Mixt	Cutaneous, Oral	ash	4	3
nd	*Oryza sativa* L.	Loso	Poaceae	Seed	Grinding, calcined	Oral	caramel, sugar	4	4
MBA/12	*Passiflora edulis* Sims	Marakuja	Passifloraceae	Fruit, FL	EJ, Dec	Oral	soft drink, sugar	2	4
MBA/18	*Persea americana* Mill.	Avocati, Isandu	Lauraceae	FL, Fruit, Pit, Bark	Dec, Mac	Oral, Cutaneous	*Citratus leaves,* sugar milk, tomato juice, caramel, ash, soft drink, eggs, sugar, baking powder	21	17
MBA/14	*Ricinus communis* L.	Mbalika	Euphorbiaceae	FL	Heating	Cutaneous	nd	1	1
MBA/03	*Senna alata* (L.) Roxb.	Folele	Fabaceae	FL	Dec	Oral	Sugar	1	2
MBA/02	*Sida acuta* Burm.f.	Omongo, Uende ukamuita mama	Malvaceae	Stem, Root	Grinding and calcined, Dec	Oral	vegetable salt palm oil ash	2	2
nd	*Solanum betaceum* Cav.	Damudamu	Solanaceae	FL, Fruit	Dec, EJ	Oral	Sugar, eggs	2	2
nd	*Solanum lycopersicum* L.	Tomate	Solanaceae	Fruit	Mixt	Oral	Soft drink	2	3
MBA/32	*Tectona grandis* L.f.	Tec	Verbenaceae	FL	Mac, Dec	Cutaneous	Tomato juice, eggs, sugar soft drink, sorghum flour, caramel,	20	12
MBA/15	*Terminalia catapa* L.	Madamé	Combretaceae	FL	Dec	Cutaneous	caramel	4	3
MBA/27	*Theobroma cacao* L.	Cacao	Malvaceae	FL, DLT	Dec	Oral	ash, sugar	4	3
MBA/31	*Zingiber officinal* Roscoe	Tangawisi	Zingiberaceae	Root	Inf	Oral	nd	1	3

Legend: Dec (Decoction), DF (Dried fruit), DLT (Dried leaves on the tree), EJ (Expression of juice), FC (Frequency of citation), FL (Fresh leaves), Inf (Infusion), Mac (Maceration), nd (not defined), Soft drink (Coca cola), Sugar milk (Nestlé), VN (vernacular name); WP (whole plant)

**Table 3:** IC_50_ of samples for antioxidant test.

Sample	Antioxidant Expressed as IC_50_ (in µg/mL) Mean ± Standard Deviation (*n* = 3), IC = 95%
*Alchornea cordifolia* (Schumach.) Müll.Arg.	
AC arid soil	1.76 ± 0.17
with caramel	nd
with ashes	12.46 ± 4.51
with lemon juice	2.89 ± 0.26
with sugar	nd
AC marsh soil	3.11 ± 0.10
with caramel	nd
with ashes	36.04 ± 25.15
with lemon juice	4.99 ± 3.59
with sugar	nd
*Hibiscus tiliaceus L.*	
HT arid soil	18.71± 11.78
with caramel	nd
with ashes	nd
with lemon juice	nd
with sugar	nd
HT marsh soil	4.85 ± 0. 86
with caramel	nd
with ash	26.22 ± 10.02
with lemon juice	12.24 ± 3.51
with sugar	nd

## Data Availability

The original contributions presented in this study are included in the article. Further inquiries can be directed to the corresponding author(s).
